# Reduced humoral but stable cellular SARS-CoV-2-specific immunity in liver transplant recipients in the first year after COVID-19

**DOI:** 10.1371/journal.pone.0276929

**Published:** 2022-11-02

**Authors:** Theresa Kirchner, Sophia Heinrich, Agnes Bonifacius, Bastian Engel, Louisa Ruhl, Isabell Pink, Nele Thomas, Joerg Martens, Marius M. Hoeper, Rainer Blasczyk, Heiner Wedemeyer, Elmar Jaeckel, Yang Li, Christine S. Falk, Britta Eiz-Vesper, Richard Taubert

**Affiliations:** 1 Department of Gastroenterology, Hepatology and Endocrinology, Hannover Medical School, Hannover, Germany; 2 Institute of Transfusion Medicine and Transplant Engineering, Hannover Medical School, Hannover, Germany; 3 Institute of Transplant Immunology, Hannover Medical School, Hannover, Germany; 4 Department of Pneumology, Hannover Medical School, member of the German Centre for Lung Research (DZL), Hannover, Germany; 5 Institute of Biostatistics, Hannover Medical School, Hannover, Germany; 6 Department for Liver Transplantation at University Health Network of the University of Toronto, Toronto, Canada; 7 Centre for Individualised Infection Medicine and TWINCORE, Joint Ventures between the Hannover Medical School and the Helmholtz Centre for Infection Research, Hannover, Germany; University of the Witwatersrand, SOUTH AFRICA

## Abstract

Mortality due to COVID-19 is not increased in immunosuppressed individuals after liver transplantation (OLT) compared to individuals without immunosuppression. Data on long-term protective immunity against SARS-CoV-2 in immunosuppressed convalescents, is limited. We prospectively measured immune responses against SARS-CoV-2 by quantifying antibodies against 4 different antigens (spike protein 1 and 2, receptor binding domain, nucleocapsid) and T cell responses by IFN-γ ELISPOT against 4 antigens (membrane, nucleocapsid, spike protein 1 and 2) in 24 OLT convalescents with immunosuppressive therapy longitudinally in the first year after COVID-19 including a booster vaccination in comparison to a matched cohort of non-immunosuppressed convalescents (non-IS-Con). Pre-pandemic OLT samples were retrieved from our prospective OLT biorepository (n = 16). No relevant T cell reactivity or immunoglobulin G (IgG) against SARS-CoV-2 were detectable in pre-pandemic samples of OLT recipients despite reactivity against endemic corona-viruses. OLT convalescents had a lower prevalence of IgG against nucleocapsid (54% vs. 90%) but not against spike protein domains (98–100% vs. 100%) after vaccination in the second half-year after COVID-19 compared to non-IS-Con. Also, concentrations of anti-nucleocapsid IgG were lower in OLT convalescents than in non-IS-Con. Concentration of IgG against spike protein domains was significantly increased by a booster vaccination in OLT convalescents. But concentration of IgG against two of three spike protein domains remains slightly lower compared to non-IS-Con finally. However, none of these differences was mirrored by the cellular immunity against SARS-CoV-2 that remained stable during the first year after COVID-19 and was not further stimulated by a corona vaccination in OLT convalescents. In conclusion, despite lower concentrations of anti-SARS-CoV-2 IgG in OLT convalescents anti-SARS-CoV-2 cellular immunity was as robust as in non-IS-Con.

## Introduction

Coronavirus Disease-2019 (COVID-19), caused by Severe acute respiratory syndrome Coronavirus 2 (SARS-CoV-2), is the most severe viral pandemic since the introduction of solid organ transplantation (SOT), causing hundreds of thousands of deaths and stressing health care systems all over the world.

Although patients compromised by age and comorbidities exhibit the highest morbidity and mortality, immunosuppression resulting after orthotopic liver transplantation (OLT) or other SOTs is not generally associated with a more severe course of COVID-19 [1–4]. While one, but not all, retrospective multicenter studies showed a higher need for invasive ventilation in OLT patients, but there was no increased overall mortality of OLT patients [2,3]. In this context, fatal outcome of COVID-19 was associated with more or less the same risk factors (age and comorbidities) as in non-transplanted control cohorts [1–3,5]. While a first report from Spain [2] described a negative influence of mycophenolate, especially in higher doses (> 1000 mg/day), this was not confirmed by another international study, which found a beneficial effect of tacrolimus in OLT recipients [1].

First studies demonstrated the development of an initially delayed but otherwise robust humoral and cellular immune response against SARS-CoV-2 in immunosuppressed SOT recipients compared to immunocompetent non-immunosuppressed convalescents (non-IS-Con) during and early after COVID-19 [6–11]. Only some studies showed a lower seroconversion rate of antibodies against SARS-CoV-2 in SOT compared to non-IS-Con during COVID-19 [12]. However, antibodies against SARS-CoV-2 nucleocapsid protein declined faster in immunosuppressed SOT in comparison to non-IS-Con in the first months after COVID-19 [13]. In addition, the presence of antibodies against nucleocapsid rather than against the spike protein was associated with SARS-CoV-2 neutralization in convalescent OLT recipients [14]. However, humoral immunity is only one defense mechanism of the infection immunity. The data on cellular immune responses against SARS-CoV-2, especially longitudinally over time, are limited.

The aim of the current study was to assess the humoral and cellular immune response in COVID-19 convalescent OLT recipients with ongoing immunosuppressive therapy throughout the first year after COVID-19, in order to provide insights into whether immunosuppressive therapy affects the establishment of SARS-CoV-2 immunity in these patients.

## Material and methods

### Patients and study outline

**Fig 1:**
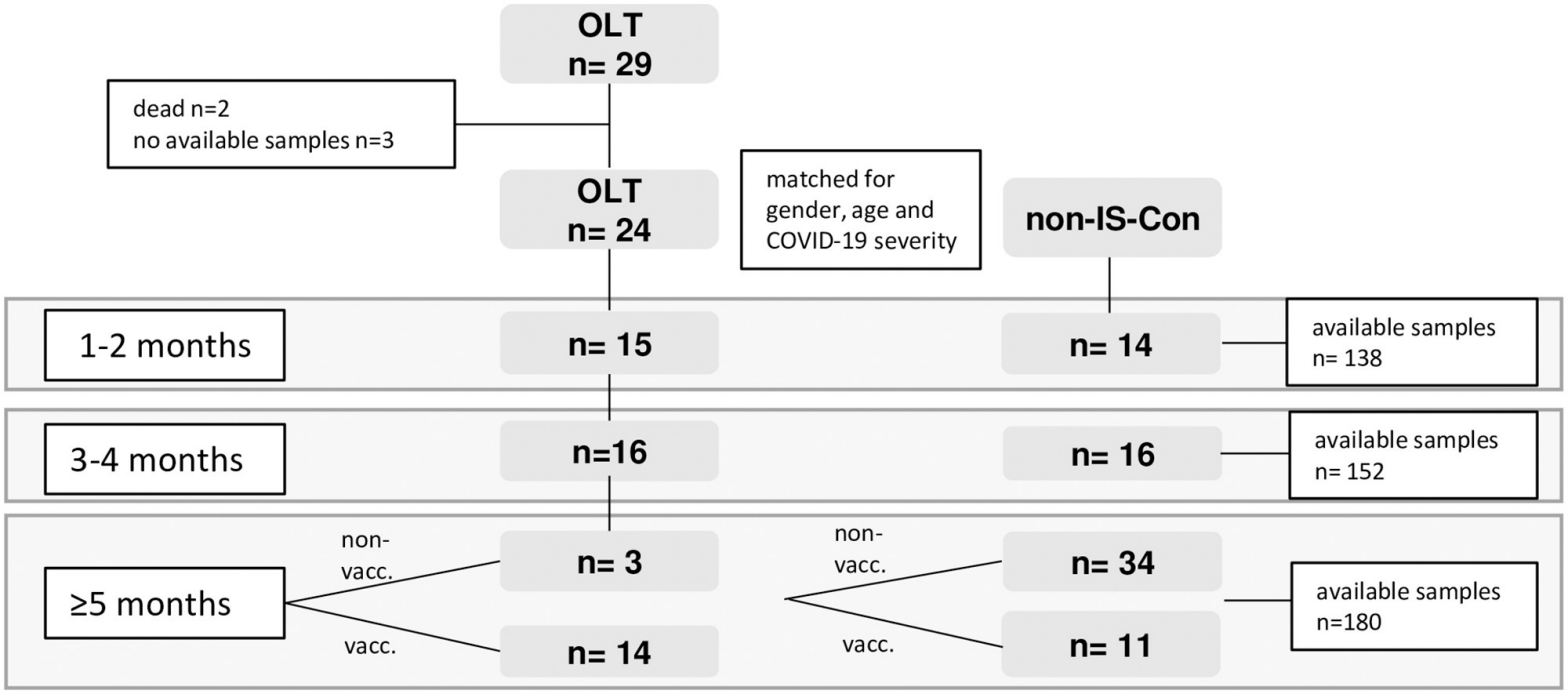
Flow chart of patient selection and study design.

All patients under immunosuppressive therapy after liver transplantation, who informed our liver transplant center about their COVID-19 disease and agreed to participate in this prospective observational study were included (Fig 1). Diagnosis was made by a positive SARS-CoV-2 RT-PCR swab testing of the upper respiratory tract (nose or throat) in the first days of active COVID-19. Patients with active COVID-19 were managed as outpatients per regular telephone calls and were recruited during the first outpatient visit after the end of their quarantine period or during hospitalization in our center. We had no information on patients’ virus strains in this study. Cryo-conserved plasma and PBMC samples of OLT patients were retrieved from our prospective OLT biorepository. To exclude an exposure to SARS-CoV-2, only samples stored before the year 2019 were used for this study. The study was approved by the local research Ethics Committee of Hannover Medical School. Written informed consent was obtained from all patients from the prospective biomaterial repository of Hannover Medical School for OLT patients (approval numbers 5582 and 933 for project Z2 of comprehensive research center 738).

While immunosuppressed convalescents after liver transplantation (OLT) were followed-up longitudinally, non-immunosuppressed convalescents without transplantation (non-IS-Con) were selected from a cross-sectional cohort matched to gender, age and COVID-19 severity of OLT convalescents at each time interval in the first year after COVID-19.

Convalescent individuals without immunosuppressive therapy (non-IS-Con) managed at our center [15] were selected as non-immunosuppressed comparators from an already existing biorepository in a cross-sectional manner. Non-IS-Con patients were matched as far as possible to the immunosuppressed OLT cohort in terms of age, gender and COVID-19 severity in each time interval after COVID-19. The utilization of non-IS-Con was approved by the Internal Review Board of Hannover Medical School (MHH, approval number 3639_2017, 9001_BO-K, 9255_BO_K_2020, 9226_BO_K_2020), and all donors were recruited from MHH. PBMCs from non-IS-Con were isolated from either EDTA whole blood samples or residual blood samples from platelet and plasma apheresis disposables used for routine collection.

Plasma and serum samples were obtained from EDTA and serum collection tubes, respectively, and stored at -20°C until further use.

All methods were performed in accordance with the relevant guidelines and regulations.

### Detection of antiviral T cells by IFN-γ ELISPOT

SARS-CoV-2-specific T lymphocytes were detected by IFN-γ ELISPOT assay as previously described [15]. Briefly, PBMCs were isolated by discontinuous density gradient centrifugation, resuspended in culture medium (CM) consisting of RPMI-1640 (Lonza, Vervies, Belgium) supplemented with 10% human AB serum (C.C.pro, Oberdorla, Germany) at a concentration of 1x10^7^ cells/mL, seeded in 24-well plates and rested overnight. Rested PBMCs were cultured in anti-IFN-γ pre-coated ELISPOT- plates (Lophius Biosciences, Regensburg, Germany) for 16–18 hours at a density of 2.5x10^5^ cells/well with specific antigens of interest. Overlapping peptide pools against SARS-CoV-2 membrane (M), nucleocapsid (N) and spike (S vial1, S vial2) proteins as well as a peptide pool containing a selected mixture of peptides derived from different SARS-CoV-2 proteins (Select) (Miltenyi Biotec, Bergisch Gladbach, Germany and JPT, Berlin, Germany) were used to stimulate each sample. Antigens of the S1 and S2 epitopes of endemic coronavirus strains HCoV229E and HCoVOC43, antigens derived from human Respiratory Syncytial Virus (RSV, nucleoprotein) and Influenza A Virus (IAV, matrix protein 1, MP1; all supplied by JPT, Berlin, Germany) were also analyzed. Pools were used at a final concentration of 1 μg of each peptide/mL. Cells stimulated with staphylococcal enterotoxin B (1 mg/mL, SEB, Merck, Taufkirchen, Germany) served as positive controls, and unstimulated PBMCs as negative controls (NC). IFN-γ secretion was detected using streptavidin-alkaline phosphatase (Mabtech, Stockholm, Sweden) and revealed by 5–13 bromo-4-chloro-3-indolyl phosphate and nitroblue tetrazolium (BCIP/NBT Liquid Substrate, Merck, Darmstadt, Germany). Spots were counted using AID ELISPOT 8.0 on an AID iSpot spectrum reader system (both from AID, Strassberg, Germany). Mean values of duplicate well readings were calculated and expressed as the number of spots per well (spw). PBMCs were stained with anti-CD45 APC-H7, anti-CD3 FITC, anti-CD4 PerCP and anti-CD8 APC (BD Biosciences, San Jose, USA), acquired at BD FACSCanto 10c and analyzed using BD FACSDiva Software version 8.0.1 for calculation of spots/10,000 CD3^+^ T cells (both from BD Biosciences, San Jose, USA).

### Detection of anti-SARS-CoV-2 antibodies

SARS-CoV-2-specific antibodies were detected using the SARS-CoV-2 Antigen Panel 1 IgG assay (Millipore HC19SERG1-85K-04, Merck Taufkirchen, Germany) following the manufacturer’s instructions. Plasma samples were diluted 1:200 with sample diluent. The semi-quantitative readout was given as median fluorescence intensity (MFI) of > 50 beads for each antigen and sample, acquired by the Bio-Plex 200 machine and the Bio-Plex Manager^™^ Version 6.0 software (Bio-Rad, Hercules, USA).

### Statistical analysis

Statistical analysis was performed with SPSS 15.0 (IBM, Armonk, USA). Descriptive statistics were calculated for the OLT and the non-IS group. Mann-Whitney U tests were used for the comparison of two groups. Longitudinal comparison of paired samples was compared using the paired Wilcoxon rank sum test. Chi^2^ test and Fisher’s exact test, if Chi^2^ was not possible, were used to compare contingency tables. P-values below 0.05 (two-tailed) were considered statistically significant in all analyses.

## Results

### Patient characteristics and COVID-19 disease course after OLT

Twenty-nine OLT patients informed our center about their COVID-19 (Fig 1). Recruitment for this prospective oberservational studystarted in December 2020 and as early as four weeks after end of isolation, meaning approximately six weeks after diagnosis of SARS-CoV-2 infection during a patient visit at our transplantation center. Recruitment at later time points after COVID-19 usually resulted from delayed notification of the SARS-CoV-2 infection/COVID-19 by the patients before the start of this study. A study visit was offered to all OLT recipients every three months in the first year after COVID-19. Plasma and/or PBMC samples before the SARS-CoV-2 pandemic, stored in 2019 and earlier, were available from our biorepository in 16/24 (67%) OLT convalescents. All patients were already under immunosuppression after OLT when plasma was stored.

Since begin of pandemic until end of study (03/22), two OLT patients died after the diagnosis of COVID-19. One OLT recipient with an already established re-cirrhosis died during hospitalization for COVID-19 before recruitment into this study. One patient with critical disease severity died three months after COVID-19 from a cholangiosepsis due to a secondary sclerosing cholangitis, which the patient acquired during intensive care for COVID-19 including ECMO therapy (Fig 1). Both these patients died before written informed consent for this study. Three patients were excluded due to a lack of available biomaterials (Fig 1).

In total 24 OLT recipients (46% female) with documented COVID-19 between 11/2020 and 05/2021 were recruited into this prospective study in median 4.5 years after OLT (Fig 1, Table 1).

**Table 1:** Demographic and clinical data of convalescents after liver transplantation.

**OLT (n = 24)**					
**Sex**	male (n;%)	13; 54.2			
**Time after transplantation in years**	median (min; max)	4.5 (1;28)			
**Underlying liver disease**					
Hereditary	n; %	7; 29			
Autoimmune liver disease	n; %	6; 25			
Viral hepatitis	n; %	5; 21			
Acute liver failure	n; %	2; 8.3			
Cryptic cirrhosis	n; %	2; 8.3			
Metabolic liver disease	n; %	2; 8.3			
**Immunosuppression at diagnosis of COVID-19**					
Steroids	yes n (%)	9; 27.5			
Tacrolimus	low n (%)	6; 25			
	high n (%)	15; 62.5			
	no Tac n (%)	3; 12.5			
CsA	yes n (%)	3; 12.5			
CNI	yes n (%)	24; 100			
MMF	yes n (%)	18; 75			
MMF dose in mg/day	median (min; max)	500 (0; 1500)			
Everolimus	yes n (%)	3; 12.5			
Aza	yes n (%)	1; 4.2			
Aza dose in mg/day		50			
Sirolimus	yes n (%)	1; 4.2			
**IS score**	1	1; 4.2			
	2	8; 33.3			
	3	6; 25			
	4	6; 25			
	5	2; 8			
	6	1; 4.2			
**Reduction immunosuppression during COVID-19**	yes (n;%)	4; 16.6			
**Symptoms during infection (multiple answers possible)**					
None	n; %	2; 13.3			
Cough/dyspnea	n; %	9; 60			
Fatigue	n; %	5; 30			
Fever	n; %	4; 26.7			
Body aches	n; %	3; 20			
Headache	n; %	2; 13.3			
Score throat	n; %	2; 13.3			
Sweat	n; %	1; 6.7			
Sniffles	n; %	1; 6.7			
**WHO clinical progression scale**					
	1	7			
	2	14			
	3	1			
	4	2			
**Hospitalization during COVID-19**	yes (n;%)	4; 16.6			
		**1–2 months**	**3–4 months**	**≥ 5 months non-vacc.**	**≥ 5 months vacc.**
		n = 15	n = 16	n = 3	n = 14
**Sex**	male (n;%)	9; 60	9; 56.3	2; 66.7	8; 57.1
**Age**	median (min; max)	56 (25;68)	56 (22;72)	56 (20;68)	56 (32; 72)
**Time after diagnosis in weeks**	median (min; max)	6 (3;8)	15 (9;20)	26 (25; 30)	29 (24;56)
**Symptoms (multiple answers possible)**					
None	n; %	3; 20	10; 62.5	3; 100	11; 78.6
Cough/dyspnea	n; %	4; 26.7	3, 18.8	0; 0	1; 12.8
Fatigue	n; %	6; 40	2; 12.5	0; 0	2; 14.3
Fever	n; %	1; 6.7	0; 0	0; 0	0; 0
Body aches	n; %	1; 6.7	0; 0	0; 0	0; 0
Headache	n; %	1; 6.7	0; 0	0; 0	0; 0
Score throat	n; %	3; 20	2; 12.5	0; 0	0; 0
Sweat	n; %	0; 0	0; 0	0; 0	0; 0
Sniffles	n; %	1; 6.7	0; 0	0; 0	0; 0
AST	median (min; max)	25 (8;180)	26 (15;100)	20 (13;22)	26 (16;68)
ALT	median (min; max)	28 (10;308)	25 (5;86)	15 (12;21)	22 (4;82)
GGT	median (min; max)	32 (11;1353)	32 (5;487)	19 (18;86)	26 (7;584)
AP	median (min; max)	104 (58;564)	92 (60;470)	99 (60;110)	98 (5;630)
Bili	median (min; max)	9 (5;74)	13 (5;23)	6 (4;10)	12 (7;118)
IgG	median (min; max)	10.37 (7.7; 22.35)	10.54 (6.02; 18.61)	10.95 (10.95; 10.95)	10.41 (9.5; 18.85)

All included OLT convalescents had a mild or moderate COVID-19 severity according to WHO clinical progression scale [16], and most were managed as outpatients (20/24).

Immunosuppression was CNI-based in all OLT patients (Tacrolimus 87.5%, Cyclosporine A 12.5%) and combined with mycophenolate in 75% and/or with steroids in 28% of patients (Table 1). Immunosuppression was reduced after careful risk assessment including past surveillance biopsies in our protocol biopsy program [17] in 17% of the patients. Symptoms of long COVID-19 such as dyspnea or cough as well as fatigue were reported from 12–14% of patients even beyond five months after COVID-19.

One OLT patient had elevated liver enzymes in the course of sepsis without histological evidence of rejection and later died from cholangiosepsis. Liver function tests and liver stiffness measurements were not suggestive for increased alloreactivity in OLT patients at any study visit but were rather caused by cholestatic complications, most of all non-anastomotic strictures, that had already manifested before COVID-19. Donor specific antibody (DSA) rates of 29% in OLT convalescents did not suggest further humoral allo-sensitization, since the DSA background ranges between 25 and 30% at our center [18].

Non-IS-Con were selected from a cross-sectional biorepository with already available measurements of cellular immunity against SARS-CoV-2 [15] because longitudinally paired blood samples from non-IS-Con were not available. Non-IS-Con were matched to OLT IS-con in terms of age, gender and COVID-19 severity in each time interval after COVID-19 (Table 2).

**Table 2:** Demographic and clinical data of convalescents after liver transplantation and matched non-immunosuppressed comparators.

		Pre-pandemic	1–2 months p			3–4 months p			≥ 5 months non-vacc p.			≥ 5 months vacc. p		
		OLT (n = 16)	OLT (n = 15)	non-IS-Con (n = 14)		OLT (n = 16)	non-IS-Con (n = 16)		OLT (n = 3)	non-IS-Con (n = 34)		OLT (n = 14)	non-IS-Con (n = 11)	
Sex	male (n;%)	10; 62.5	9; 60	8; 57.1	.881	9; 56.3	10; 62.5	.729	2; 66.7	25; 74	.858	8; 57.1	4; 36.4	.322
Age	median (min; max)	56 (20;68)	56 (25;68)	51 (32; 60)	.649	56 (22;72)	46 (21;68)	.780	56 (20;68)	50 (27;57)	.906	56 (32; 72)	50 (27;57)	.162
Time after diagnosis in weeks	median (min; max)		6 (3;8)	6 (4;8)	.707	15 (9;20)	12 (9;17)	.730	26 (25; 30)	59 (36,65)	.154	29 (24;56)	59 (36;65)	.000
Inpatient	yes (n;%)		2; 13.3	0; 0	.678	3; 18	0; 0	.715	2; 66.7	0; 0	.953	1; 7.1	0; 0	.088
WHO clinical progression scale	1		3	1		3	1		1	0		6	2	
	2		11	12		11	14		1	33		7	7	
	3		0	0		1	1		0	1		1	0	
	4		1	0		1	0		1	0		0	2	

(p-values compare OLT and non-IS-Con at the time point after COVID-19 disease, pre-pandemic samples are not compared to another group).

14 OLT convalescents were vaccinated during the follow up period (Fig 1), no severe side effects were reported (mRNA-1273 (Moderna) 21,4%, BNT162b2 (Pfizer) 50%, AZD1222 (Astra) 28,6%).

### SARS-CoV-2-specific humoral immunity in immunosuppressed OLT convalescents

OLT patients exhibited no relevant deficiency of immunoglobulin G (IgG) that might bias the measurements of the humoral immunity at any study visit (Table 1). Anti-SARS-CoV-2 antibodies were quantified as antibody frequencies, meanings concentrations above the reference cut-off and, antibody concentrations were expressed as median fluorescence intensity (MFI).

**Fig 2:**
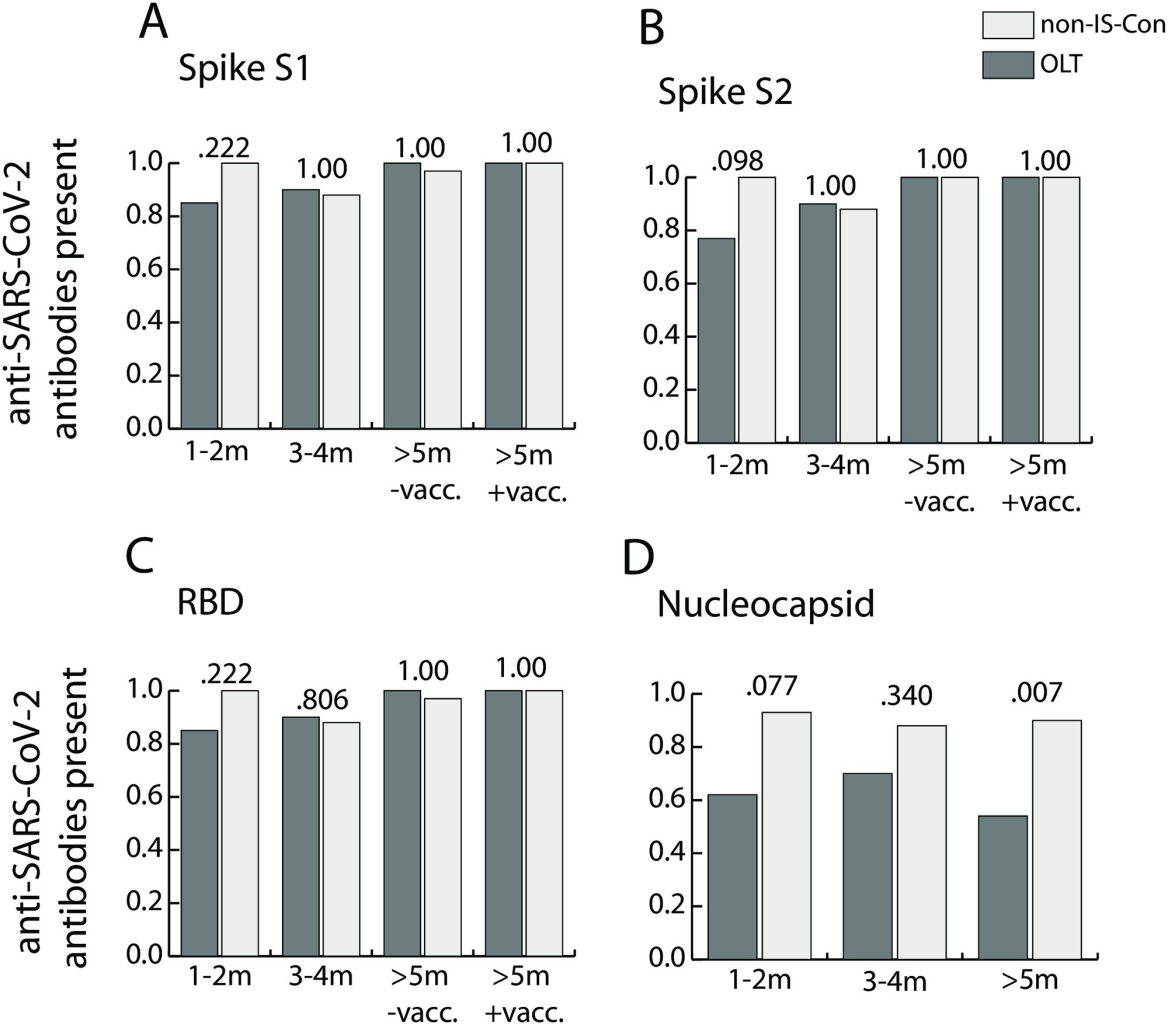
Longitudinal prevalence of SARS-CoV-2-specific immunoglobulin G. Prevalence of IgG antibody concentrations above the reference threshold against various spike protein domains (A, B) including the receptor binding domain (RBD; C) and against the nucleocapsid protein (D) in convalescents after liver transplantation (OLT; light grey) and matched non-immunosuppressed convalescents without transplantation (non-IS-Con, dark grey) in the first year after COVID-19 (m = months; vacc. = vaccination).

No anti-SARS-CoV-2 IgG antibody concentrations above the reference threshold were detectable in 15 available pre-pandemic plasma samples from OLT convalescents from our prospective biorepository (Table 2). Frequencies of IgG antibodies against various epitopes of SARS-CoV-2 spike protein (S1, S2 and receptor binding domain (RBD)) and nucleocapsid (N)) were not significantly different between OLT convalescents and non-IS-Con in the first months, at 1–2 months and 3–4 months, after COVID-19 (Fig 2A–2D). There was a non-significant trend towards lower frequencies of anti-nucleocapsid IgG antibodies in OLT convalescents compared to non-IS-Con in the first phase, 1–2 months, (p = 0.077, Fig 2D). In the later phase, ≥ 5 months after COVID-19, anti-nucleocapsid IgG antibodies were found in significantly fewer OLT convalescents (7/13; 54%) than in non-IS-Con (38/42; 90%) (p = 0.007; Fig 2D).

In contrast to anti-nucleocapsid antibodies, antibodies against the spike protein can be induced by the currently available SARS-CoV-2 vaccinations, that were recommended at six months after COVID-19. The majority of OLT convalescents (14/17, 82%) were already vaccinated at the latest study visit ≥ 5 months after COVID-19 (Table 2). Frequencies of IgG antibodies against spike protein epitopes were similar in vaccinated OLT convalescents (11/11; 100%) and vaccinated non-IS-Con (8/8; 100%) (Fig 2A–2C). In addition, anti-spike protein IgG antibodies were found in both available unvaccinated OLT convalescents at the latest time point (Fig 2A–2C).

**Fig 3:**
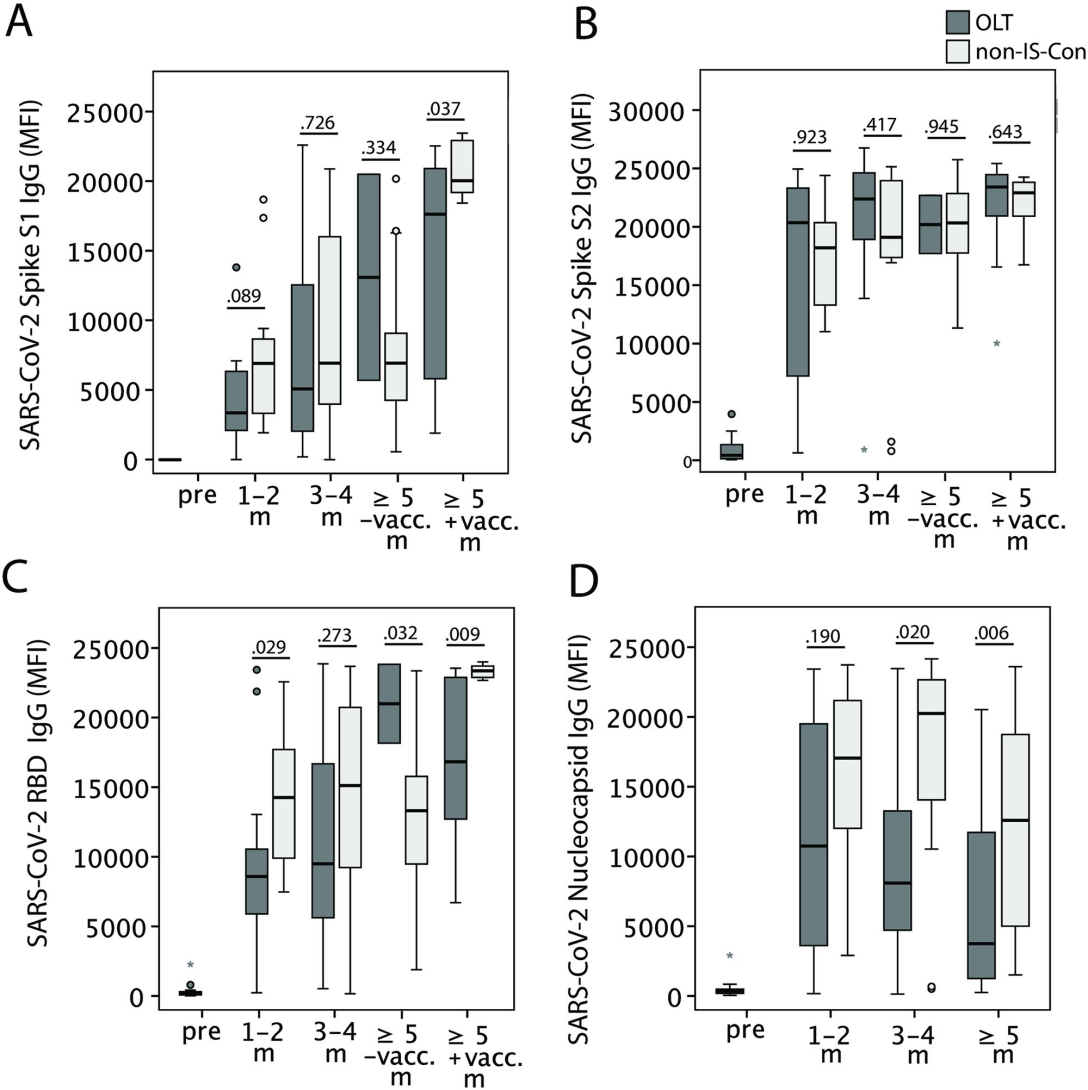
Longitudinal concentration of SARS-CoV-2-specific immunoglobulin G. Concentration of IgG antibodies, expressed as MFI, against various spike protein domains (A, B) including the receptor binding domain (RBD; C) and against the nucleocapsid protein (D) in convalescents after liver transplantation (OLT; dark grey) and matched non-immunosuppressed convalescents without transplantation (non-IS-Con, light grey) before the corona pandemic (pre) and in the first year after COVID-19 (m = months; vacc. = vaccination).

When comparing the antibody concentration expressed as MFI between OLT convalescents and non-IS-Con, concentrations of IgG antibodies against S1 domains of the spike protein were not significantly different at the first two time points after COVID-19. However, after vaccination at the last time point, OLT convalescents had lower anti-spike 1 IgG antibody concentrations compared to non-IS-Con but above the reference threshold (Fig 3A). Concentrations of IgG antibodies against S2 domains of the spike protein were not significantly different between OLT convalescents and non-IS-Con at any visit after COVID-19 (Fig 3B). Concentrations of IgG antibodies against the RBD of the spike protein were found with lower concentrations early (1–2 months), with similar concentrations later (3–4 months) and with lower concentrations after vaccination late (≥ 5 months) after COVID-19 in OLT convalescents compared to non-IS-Con (Fig 3C). In contrast, concentration of anti-nucleocapsid IgG antibodies was not different between OLT convalescents and non-IS-Con early but significantly lower later (≥ 3 months) after COVID-19 (Fig 3D).

When anti-SARS-CoV-2 antibody concentrations were assessed longitudinally in directly paired samples of OLT convalescents, the concentrations dramatically increased during COVID-19 compared to pre-pandemic samples and showed no significant longitudinal decline thereafter (S1 Table). Moreover, antibody concentrations against spike protein domains could be further increased by the booster vaccination compared with the last available time point before the vaccination (S1 Table). Unfortunately, the number of longitudinally available antibody measurements was too small for the application of a robust mixed model to compensate for missing values. Furthermore, our cohort was too small for a robust analysis of factors influencing anti-SARS-CoV-2 antibody concentrations longitudinally.

### SARS-CoV-2-specific cellular immunity in immunosuppressed OLT convalescents

**Fig 4:**
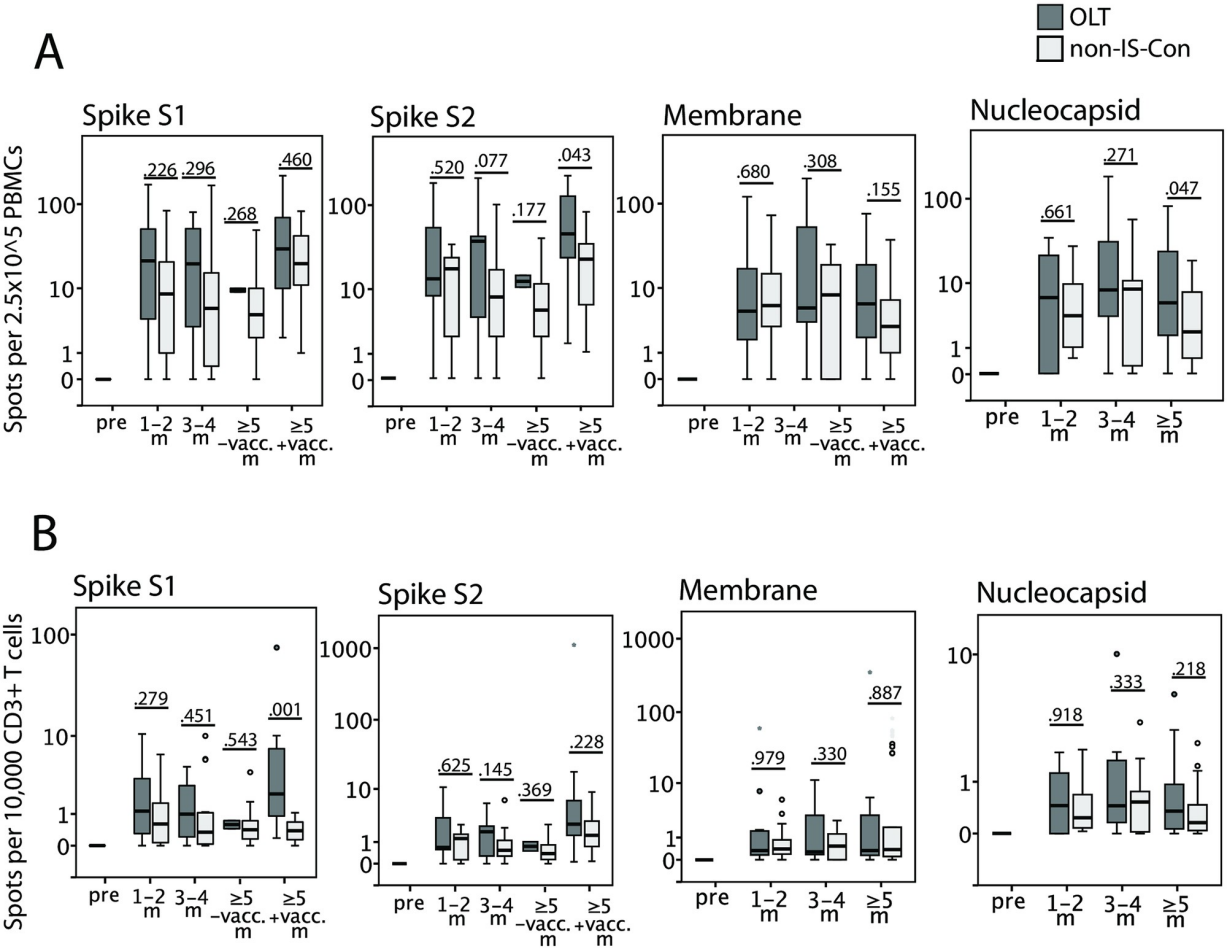
Longitudinal SARS-CoV-2-specific cellular immunity. Cellular immunity was quantified by IFN-γ production upon stimulation with various SARS-CoV-2 peptide pools in ELISPOT assays normalized to numbers of circulating PBMCs (A) as well as CD3^+^ T cells (B) in immunosuppressed COVID-19 convalescents after liver transplantation (OLT, dark grey) compared to matched non-IS convalescents (non-IS; light grey) before the corona pandemic (pre) and in the first year after COVID-19 (m = months; vacc. = vaccination).

OLT convalescents had no reduced specific T cell frequencies, normalized to numbers of PBMCs and CD3^+^ T cells, against all SARS-CoV-2 antigens tested (membrane, nucleocapsid and spike protein) in ELISPOT assays, compared to non-IS-Con at any study time point after COVID-19 (Fig 4). Especially, the lower concentrations of anti-nucleocapsid antibodies in OLT convalescents were not paralleled by a decline of cellular immunity against nucleocapsid. In addition, cellular immune response was measured even higher in OLT convalescents than in non-IS-Con at later time points after COVID-19.

When the cellular immune response was compared in OLT convalescents longitudinally, the data set was too small to apply a mixed model to compensate for missing values. Cellular reactivity against SARS-CoV-2 was not detectable in the four pre-pandemic cryo-conserved PBMC samples but increased after COVID-19. Probably due to the small sample size these comparisons to pre-pandemic samples did not reach significance (S1 Table). Cellular immunity against SARS-CoV-2 was stable at all study visits, when compared in directly paired samples (S1 Table). While concentrations of antibodies against spike protein domains could be increased by a booster vaccination, specific T cell frequencies were not further increased by the vaccination (S1 Table).

Our longitudinal data set was too small for a robust analysis of factors being associated with cellular immunity against SARS-CoV-2.

A recent study demonstrated a decline in general T cell functionality during active COVID-19 [15]. Therefore, cellular immune responses against various other respiratory viruses were assessed similarly. OLT convalescents had no reduced IFN-γ production, again normalized to PBMCs and CD3^+^ T cells against endemic coronaviruses (HCoV-OC43 and HCoV-229E), respiratory syncytial virus (RSV) and Influenza A (S1 Fig), indicating no overall reduced immune memory for other common airway viruses.

## Discussion

Measurable immune responses against SARS-CoV-2 decline over time in immunocompetent COVID-19 convalescents in the majority of studies. IgG antibodies decline mostly in the first six months and remain stable between six to twelve months after COVID-19. Cellular immune responses of T cells against SARS-CoV-2 decline later (beyond six months after COVID-19) and memory B cell response is more long-lasting than T cell memory and stable within the first year after COVID-19 [19–21].

The majority of studies, despite the heterogeneity of the study concepts, showed that immunosuppressed SOT recipients developed a humoral and cellular immune response against SARS-CoV-2 as robust as in non-IS-Con when assessed in the first weeks after COVID-19 [6–12]. However, antibodies against nucleocapsid antibodies were less frequent and were present in lower concentrations in immunosuppressed SOT recipients including liver transplanted patients compared to matched non-IS-Con in some studies [12,14]. The relevance of this finding was underlined by a recent study showing an association of SARS-CoV-2 neutralization abilities with the presence of anti-nucleocapsid antibodies in OLT convalescents [14]. In the present study, we found no significant differences in the frequencies of anti-SARS-CoV-2 IgG antibodies in the first two months after COVID-19, but with a trend towards lower IgG frequency against the nucleocapsid protein (Fig 2). When the IgG antibody concentrations were assessed, OLT convalescents had lower concentrations of IgG against the RBD than non-IS-Con (Fig 3). However, the T cell reactivity against all assessed SARS-CoV-2 peptide pools were not different between OLT convalescents and non-IS-Con (Fig 4).

A first prospective longitudinal study on humoral immunity in OLT convalescents found a lower frequency of IgG against nucleocapsid and spike protein one year after COVID-19 in 65 OLT convalescents than in propensity score-matched non-IS-Con. However, the concentration was only significantly lower for IgG against nucleocapsid but not against the spike protein in OLT convalescents compared to non-IS-Con [13,22]. A similar finding was reported in a cohort of mixed SOT convalescents compared to matched non-IS-Con [23]. The current study found a significantly lower frequency and significantly lower concentration of IgG against the nucleocapsid protein in OLT convalescents only in the long run. While antibody concentrations were not different for IgG against spike protein domains at all observation time points, the comparison of anti-spike IgG concentration was more ambiguous. This means that, while we could not observe different anti-spike IgG concentrations at the second observation time point (3–4 months after COVID-19), the concentrations of IgG against spike S1 and RBD but not against spike S2 were lower in OLT convalescents compared to non-IS-Con at the latest time point after an additional booster vaccination. This observation of our single center study was not backed by the larger Spanish multicenter study [22], which did differentiate the reactivity against the spike protein with various peptide pools as the present study. However, both studies (ours and the Spanish) observed a significant increase of anti-spike IgG concentrations after booster vaccination in OLT convalescents. The comparison of the latest time point of OLT convalescents without a booster vaccination in the current study should not be overestimated because of a low sample number (n = 2).

Due to the higher technical requirements, the data base on cellular immune responses against SARS-CoV-2 after SOT beyond the first months after COVID-19 is much smaller. At the time of writing of this manuscript, we are aware of only two longitudinal studies on cellular immunity against SARS-CoV-2 including kidney, heart and lung but no liver transplanted patients with a total number of 20 longitudinal samples [24,25]. Both studies uniformly describe stable cellular immune responses against SARS-CoV-2 including IFN-γ release as does our study but also of other cytokines (IL-2, IL-4, TNF-α etc.) and proliferation assays in SOT convalescents similar to non-IS-Con up to twelve months after COVID-19. The present study confirmed this finding of a stable and comparable cellular immunity after COVID-19 in OLT and non-IS-Con against multiple SARS-CoV-2 antigens. The lower frequency and concentration of anti-nucleocapsid IgG antibodies is not mirrored by the T cell responses detected via IFN-γ release. Interestingly, in contrast to antibody concentrations, the cellular immunity against the spike protein could not be further boosted by a vaccination in OLT convalescents. Neither of the studies mentioned above included transplanted convalescents with additional booster vaccination after COVID-19 [24,25].

A further unique aspect of the present study is the availability of pre-pandemic samples from our prospective biorepository. Unfortunately, the number of paired pre-pandemic and post-COVID-19 samples are limited to two patients for cellular and eight patients for humoral immune response measurements (S1 Table). A positive influence of a preexisting cross-reactivity on the development of a strong immunity against SARS-CoV-2, caused by exposure to endemic corona viruses (e.g. HCoV-43 and HCoV-229E), was suggested recently [15]. We could not detect preexisting IgG antibodies or T cell responses in cryo-preserved samples stored in our prospective biorepository before the appearance of SARS-CoV-2 in OLT patients. However, based on the small number of 4–12 samples, this finding suggests that robust immunity against SARS-CoV-2 can develop despite ongoing immunosuppression for SOT/OLT and mild COVID-19 severity.

A major limitation of the current study was the monocentric study design leading to a limited number of OLT convalescents. This was mostly due to the technical requirements of IFN-γ release assays with the best test results from fresh PBMC samples. Additionally, many OLT patients declined longitudinal study visits every three months after COVID-19 as intended in the initial study design. Reasons were long travel distances to our center and a general reluctance of OLT recipients to visit our center in the corona pandemic to reduce the risk of reinfections with other SARS-CoV-2 variants. The small sample number prevented further analyses e.g. correlation analysis of immunosuppression regimen with quantitative measures of cellular and humoral immune response. This would have been of relevant interest, because register studies suggested a negative impact of mycophenolate with daily dosages above 1 g or a beneficial effect of tacrolimus use on the clinical course of COVID-19 after OLT [1,2]. A further shortcoming of the current study was the lack of a longitudinal comparator cohort of non-IS-Con. These controls had to be selected from a cross-sectional non-IS-Con cohort separately for each time point. A final shortcoming of the study is that, although prospectively performed over more than one year, it only allows retrospective conclusions on the development of the immunity against previously, but already vanished, SARS-CoV-2 virus types in non-vaccinated OLT recipients. The majority of patients inthis study were infected during the first pandemic spread dominated by the wild, alpha and delta virus type but not thecurrently dominating omicron virus type. Luckily, nearly all willing OLT recipients had access to a full corona vaccination. Although the seroconversion rates and cellular response rates are not 100%, the population of immunological SARS-CoV-2 naive OLT recipients is nearly nonexistent anymore.

Multiple evaluations from COVID-19 registries of OLT patients did not report increased rejection rates after COVID-19 [1–3,5]. In this sense, we found no evidence for increased alloreactivity in terms of liver enzyme elevation, liver stiffness measurement or frequency of DSAs in our non-invasive surveillance measurement, either.

In summary, the current study confirms previously published data on lower longitudinal humoral immunity against the nucleocapsid protein in OLT convalescents compared to non-IS-Con. Although anti-spike IgG were detectable in nearly all OLT convalescents after a further booster vaccination the IgG concentrations against some spike domains especially the RBD were lower in OLT convalescents. This observation fuels the ongoing discussion on further booster vaccination to support humoral long-term immunity in SOT recipients. However, all these differences in humoral immunity were not mirrored at the level of T cell responses in OLT recipients, which were as robust as in non-IS-Con. Further long-term observations of humoral and cellular immunity against SARS-CoV-2 will be necessary to adjust our long-term strategies to prevent SARS-CoV-2 reinfection in the coming era of endemic SARS-CoV-2.

## Supporting information

S1 Fig Cellular immunity against common airway viruses in immunosuppressed early convalescents. IFN-γ production upon stimulation with antigen sets from various endemic corona viruses and other airway common viruses in ELISPOT assays normalized to numbers of circulating PBMCs (A) as well as CD3+ T cells (B) was not significantly different in immunosuppressed COVID-19 convalescents after liver transplantation (OLT, dark grey) compared to matched nonimmunosuppressed convalescents (non-IS, light grey) before the corona pandemic (pre) and in the first year after COVID-19 (m = months).
(TIF)

S1 Table Longitudinal paired comparisons of humoral and cellular immunity in OLT convalescents. (DOCX)

## Abbreviations

ALT: alanine aminotransferase
AST: aspartate aminotransferase
CNI: calcineurin inhibitor
COVID-19: Coronavirus Disease-2019
DSA: donor specific antibodies
IgG/M/A: immunoglobulin G/M/A
MFI: median fluorescence intensity
non-IS-Con: non-immunosuppressed convalescents
OLT: orthotopic liver transplantation
PBMC: Peripheral blood mononuclear cell
RBD: receptor binding domain
SARS-CoV-2: severe acute respiratory syndrome coronavirus type 2
SOT: solid organ transplantation
