# Supplementary figures and images for: Reduced humoral but stable cellular SARS-CoV-2-specific immunity in liver transplant recipients in the first year after COVID-19
Source: PLoS One. 2022 Nov 2;17(11):e0276929. doi: 10.1371/journal.pone.0276929 (PMC9629592; doi:10.1371/journal.pone.0276929)

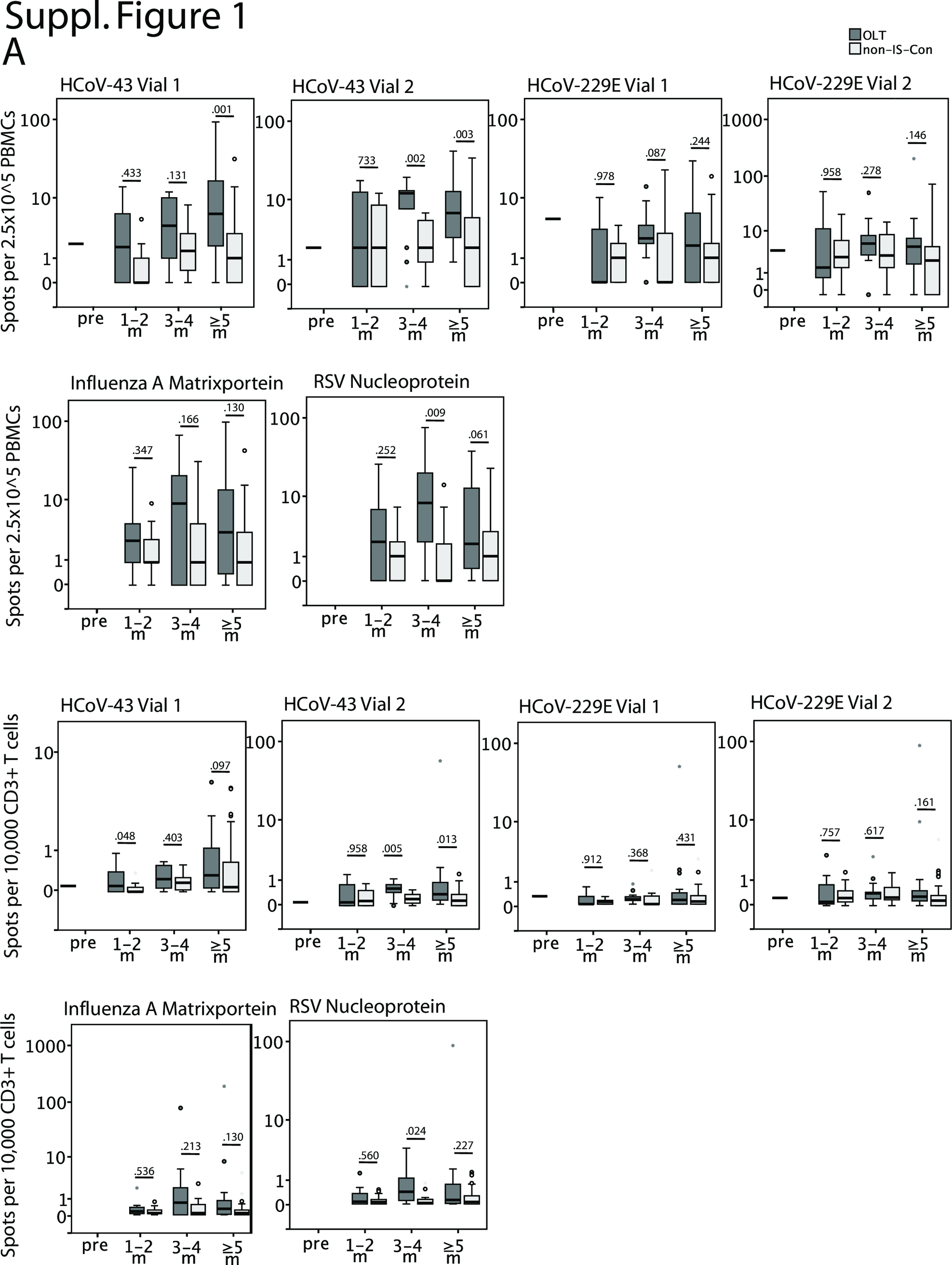

Supplement: S1 Fig — IFN-γ production upon stimulation with antigen sets from various endemic corona viruses and other airway common viruses in ELISPOT assays normalized to numbers of circulating PBMCs (A) as well as CD3+ T cells (B) was not significantly different in immunosuppressed COVID-19 convalescents after liver transplantation (OLT, dark grey) compared to matched nonimmunosuppressed convalescents (non-IS, light grey) before the corona pandemic (pre) and in the first year after COVID-19 (m = months). (TIF) [file pone.0276929.s001.tif]
