# Supplementary material for: Reduced humoral but stable cellular SARS-CoV-2-specific immunity in liver transplant recipients in the first year after COVID-19
Source: PLoS One. 2022 Nov 2;17(11):e0276929. doi: 10.1371/journal.pone.0276929 (PMC9629592; doi:10.1371/journal.pone.0276929)
Supplement: S1 Table — (DOCX) [file pone.0276929.s002.docx]

**Supplemental Table 1: Longitudinal paired comparisons of humoral and cellular immunity in OLT convalescents.**

|  | n | Humoral immunity, IgG | | | | n | Cellular immunity, Spots/10^5^PMBCs | | | | Cellular immunity, Spots/10^3^ CD3^+^ cells | | | |
| --- | --- | --- | --- | --- | --- | --- | --- | --- | --- | --- | --- | --- | --- | --- |
|  |  | Spike S1 | Spike S2 | RBD | Nucleocapsid |  | Spike S1 | Spike S2 | membrane | Nucleocapsid | Spike S1 | Spike S2 | membrane | Nucleocapsid |
| pre vs. 1-2 M | 8 | .012 | .012 | .012 | .012 | 2 | .180 | .180 | .180 | .180 | .180 | .180 | .180 | .180 |
| 1-2 M vs. 3-4 M | 8 | .025 | .050 | .263 | .484 | 7 | .866 | .933 | .176 | .248 | .169 | .735 | .866 | .249 |
| 3-4 M vs. 5 M non-vacc. | 2 | .655 | .655 | .180 |  | 2 | .655 | .180 |  |  | .189 | .180 |  |  |
| 3-4 M vs. 5 M vacc. | 4 | .144 | .144 | .144 |  | 4 | .715 | .465 |  |  | 1 | 1 |  |  |
| 3-4 M vs. 5 M (all) | 6 |  |  |  | .463 | 6 |  |  | .600 | .345 |  |  | .528 | .345 |
| pre (1-4M) vs post vacc. | 7 | .028 | .028 | .028 |  | 9 | .594 | .314 |  |  | .515 | .594 |  |  |
| 1-4 M vs. 5 M | 9 |  |  |  | .139 | 11 |  |  | .929 | .790 |  |  | .563 | .594 |

Outlined are p-values of paired Wilcoxon rank sum test of antibody concentrations outlined in figure 3.
